# Identification and Mechanistic Analysis of Toxic Degradation Products in the Advanced Oxidation Pathways of Fluoroquinolone Antibiotics

**DOI:** 10.3390/toxics12030203

**Published:** 2024-03-06

**Authors:** Shuhai Sun, Zhonghe Wang, Qikun Pu, Xinao Li, Yuhan Cui, Hao Yang, Yu Li

**Affiliations:** 1 School of Hydraulic and Environmental Engineering, Changchun Institute of Technology, Changchun 130012, China; sun18117471@163.com; 2 MOE Key Laboratory of Resources and Environmental System Optimization, North China Electric Power University, Beijing 102206, China; zhonghe_wang1999@163.com (Z.W.); puqikun2000@163.com (Q.P.); lixinao921734261@163.com (X.L.); 120222232050@ncepu.edu.cn (Y.C.)

**Keywords:** fluoroquinolones, advanced oxidation processes, degradation products, environmental and human health risk

## Abstract

The degradation of fluoroquinolones (FQs) via advanced oxidation processes (AOPs) is a promising avenue, yet the complete mineralization of certain FQ molecules remains elusive, raising concerns about the formation of toxic by-products. This study delineates five primary AOP degradation pathways for 16 commercially available FQ molecules, inferred from existing literature. Density functional theory (DFT) was employed to calculate the bond dissociation energies within these pathways to elucidate the correlation between bond strength and molecular architecture. Subsequently, Comparative Molecular Similarity Index Analysis (CoMSIA) models were constructed for various degradation reactions, including piperazine ring cleavage, defluorination, hydroxylation, and piperazine ring hydroxylation. Three-dimensional contour maps generated from these models provide a deeper understanding of the interplay between FQ molecular structure and bond dissociation energy. Furthermore, toxicity predictions for 16 FQ molecules and their advanced oxidation intermediates, conducted using VEGA 1.2.3 software, indicate that degradation products from pathways P2 and P5 pose a heightened health risk relative to their parent compounds. Furthermore, the application of the Multwfn program to compute the Fukui function for FQ molecules discerns the disparity in degradation propensities, highlighting that N atoms with higher f0 values can augment the likelihood of piperazine ring cleavage. HOMO-LUMO distribution diagrams further confirm that methoxy substitution at the 1-position leads to a dilution of HOMOs on the piperazine ring and an increased energy gap for free radical reactions, diminishing the reactivity with hydroxyl radicals. This study elucidates the pivotal role of structural characteristics in FQ antibiotics for their degradation efficiency within AOPs and unveils the underlying mechanisms of bond dissociation energy disparities. The toxicity parameter predictions for FQ molecules and their intermediates offer unique perspectives and theoretical underpinnings for mitigating the use of high-risk FQs and for devising targeted degradation strategies to circumvent the generation of toxic intermediates in AOPs through molecular structure optimization.

## 1. Introduction

Antibiotics, as critical agents in the treatment and prophylaxis of bacterial infections, are renowned for their robust antimicrobial capabilities, rapid onset, and significant therapeutic benefits, leading to their extensive deployment in healthcare and animal husbandry [1]. The proliferation of antibiotics has been instrumental in saving countless lives. However, this widespread usage has a significant environmental repercussion: the discharge of antibiotic-rich wastewater and waste from hospitals, aquaculture, pharmaceutical industries, and waste disposal processes, leading to grave environmental pollution [2]. Research has revealed that the concentration of ciprofloxacin (CIP) in surface waters can reach several hundred nanograms per liter [3], and in wastewater treatment plant effluents, concentrations can soar to 5 micrograms per liter [4]. Alarmingly, concentrations in wastewater from hospitals and pharmaceutical plants can reach milligram per liter magnitudes [5]. The persistence of FQs and their resistance to conventional biological degradation methods result in their frequent detection in various settings, posing insidious threats to ecological systems and human health [6]. Consequently, the development and implementation of efficacious treatment technologies for FQ-laden wastewater is of paramount importance.

The array of antibiotic removal methodologies encompasses coagulation, membrane separation, adsorption, and biodegradation [7]. However, these approaches often fall short in widespread application due to suboptimal removal efficiencies and prohibitive operational costs [7,8,9,10]. Advanced oxidation processes (AOPs) stand out for their efficacy in treating a wide array of recalcitrant organic pollutants, making them suitable for the decomposition of all antibiotic categories [11]. For example, biochar can be used as a catalyst to adsorb persulfate to oxidize NOR [11]. AOPs leverage potent oxidizing free radicals, such as hydroxyl radicals (HO·), singlet oxygen (^1^O_2_), and superoxide anion radicals (O^−^), to decompose complex organic molecules into simpler substances, thereby facilitating the efficient treatment of recalcitrant organic contaminants [12]. However, the performance of AOPs in degrading different FQ antibiotics is markedly variable in complex wastewater scenarios [13]. For instance, Sciscenko et al. [14] reported variable degradation rates for different FQ antibiotics using H_2_O_2_/sunlight-based AOPs, with enrofloxacin, sarafloxacin, and ofloxacin showing a descending order of degradation after 120 min. Ao et al. [15] observed that ultraviolet/peracetic acid (MPUV/PAA) processes achieved removal rates of 92.1% and 96.5% for levofloxacin and norfloxacin, respectively, under 500 mJ/cm of ultraviolet irradiation. Bobu et al. [16] reported significant differences in the removal rates of enrofloxacin and ciprofloxacin using ozone/ultraviolet-based AOPs. Thus, the structural variances of FQ antibiotics significantly influence their degradability in AOPs. Hence, the structural diversity of FQ antibiotics plays a critical role in their degradation potential during AOPs. Current research trends focus on the development of novel AOPs that are both more efficient and cost-effective, with an emphasis on increasing radical concentrations to boost degradation. However, there is a scarcity of studies that investigate the relationship between the structural characteristics of antibiotics and their degradability. Investigating this relationship could be of paramount importance for theoretically underpinning the enhancement of degradation efficiency.

While AOPs have shown efficacy in breaking down antibiotics, the limited mineralization and retention of bioactive configurations could lead to degradation products with equal or greater toxicity [1]. Li et al. [17] applied QSAR methodologies to forecast the genotoxic potential of CIP and its by-products, finding increased genotoxicity in certain intermediates compared to the original compound. Wang et al. [18] developed QSAR models with high predictive accuracy for evaluating the toxicity of FQs’ photodegradation products, identifying an increase in toxicity for some products. Wachter et al. [19] leveraged the ECOSAR model to estimate the acute and chronic toxicity of CIP and its intermediates on various aquatic organisms, uncovering potential chronic threats. Predictive modeling is thus essential for ecological and health risk assessments of FQs and their intermediates in AOPs, offering unique perspectives and theoretical backing for mitigating the use of FQs with high-risk profiles and for devising strategies to prevent the production of highly toxic intermediates by optimizing molecular structures.

While extensive research has been conducted on the environmental risks of fluoroquinolone (FQ) antibiotics, the environmental and health risks of their degradation intermediates remain underexplored. Therefore, summarizing the toxicity of these intermediates is crucial for reducing the use of FQ parent molecules. This study aims to provide insights and theoretical support for controlling the generation of less toxic degradation pathways in advanced oxidation processes (AOPs) through molecular structure analysis. The primary research goals are (1) to calculate bond dissociation energies for FQ molecules in various advanced oxidation pathways using density functional theory and to identify patterns between bond energy and molecular structure through cluster analysis; (2) to create a 3D-QSAR model of FQ molecules’ degradation capacity in AOPs using SYBYL-X2.0, delving into how molecular structure impacts bond dissociation energy; (3) to forecast 17 toxicity parameters for 16 FQ molecules and their intermediates with VEGA 1.2.3, evaluating the health and ecological risks; (4) to construct a 2D-QSAR regression model via machine learning, linking FQ degradation potential with molecular features; and (5) to employ Fukui functions and HOMO-LUMO distribution to reveal the degradation process’s bond dissociation energy mechanisms. This research will explore methods for enhancing the degradation of FQ molecules in AOPs and provide theoretical support for minimizing the use of high-risk FQ molecules and preventing the formation of highly toxic intermediates.

## 2. Materials and Methods

### 2.1. Source of FQ Molecular Structure—PubChem Retrieval Method

Existing literature reveals that 16 commercially available fluoroquinolones (FQs), including ciprofloxacin (CIP), norfloxacin (NOR), gatifloxacin (GAT), and sparfloxacin (SPA), are degradable in advanced oxidation systems (AOPs), with reaction pathways such as piperazine ring cleavage (P1), defluorination (P2), hydroxylation (P3), piperazine ring hydroxylation (P4), and decarboxylation (P5) [15,20]. Therefore, this study retrieved and obtained the 3D structures of 16 commercial FQ molecules from the “PubChem—3D Conformer” module in the National Library of Medicine database (https://pubchem.ncbi.nlm.nih.gov/ URL (accessed on 20 July 2023)) and used Gaussian 09 software to optimize the structures at the B3LYP/6-31G(d) basis set level. These optimized structures served as the initial structures for subsequent analysis of FQ degradation patterns and characteristics.

### 2.2. Characterization of the Degradation Capability of FQ Molecules in Advanced Oxidation Systems—DFT Coupled with Negative Index Calculation Method

**Figure 1:**
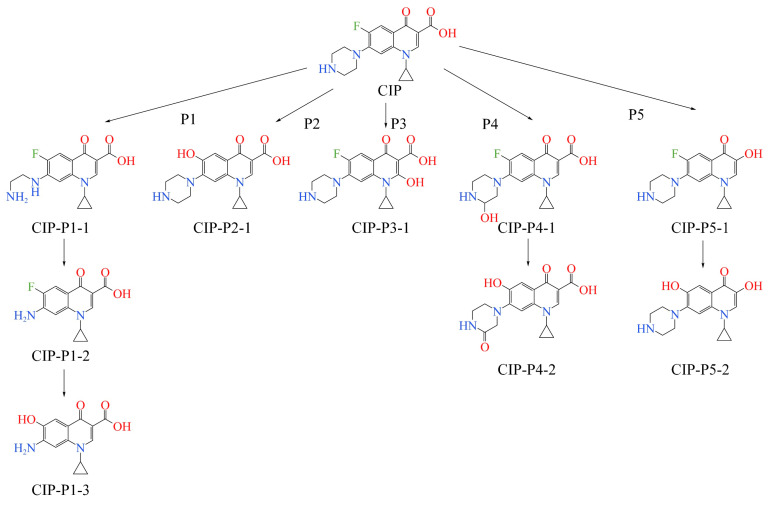
Inferred degradation pathways of FQ molecules in advanced oxidation systems (taking CIP as an example).

To comprehensively evaluate the degradation capabilities of different FQ molecules in AOPs and to analyze the degradation patterns and characteristics of different FQs, this study first searched the Web of Science literature database for the degradation methods of FQ antibiotics in AOPs, summarized the common features of FQ molecule degradation pathways in AOPs, and inferred five main advanced oxidation degradation pathways (Figure 1). Secondly, GaussView 5.0.8 software was used to draw the structures of reactants and products in the primary degradation pathways of 16 commercial FQs. Finally, based on density functional theory at the M062X/6-31G(d) basis set level [21], the Gaussian software was used to calculate the ease of primary degradation reactions of 16 FQ molecules in AOPs (characterized by the bond dissociation energy (kJ/mol) in the advanced oxidation degradation pathway). The lower the bond dissociation energy value, the easier the internal chemical bonds of the FQ molecules break, indicating higher degradability of the FQ molecules in the AOPs [22].

Given that the lower the bond dissociation energy of the FQ molecules, the easier they are degraded in the AOPs, this study employed a negative index calculation method to standardize the bond dissociation energy values of 16 commercial FQ molecules [23]. The standardization of the bond dissociation energy of FQs in AOPs is as follows:

(1) $$X_{ix}^{\prime }=\left(\max\left\{X_{ix}\right\}-X_{ix}\right)/\left(\max\left\{X_{ix}\right\}-\min\left\{X_{ix}\right\}\right)$$

where, i represents different commercial FQ molecules, i=1,2,…,16; x represents five distinct primary degradation pathways; $X_{ix}$ denotes the energy required to break a chemical bond in the i FQ molecule along the x primary degradation pathway; $min\left\{X_{ix}\right\}$ and $max\left\{X_{ix}\right\}$ represent the maximum and minimum bond dissociation energies, respectively, for the i FQ molecule in the x primary degradation pathway; $X_{ix}^{\prime }$ signifies the normalized value of the energy required to break the chemical bond of the i FQ molecule in the x primary degradation pathway.

### 2.3. Construction of 3D-QSAR Models for the Degradation Capability of FQ Molecules in Advanced Oxidation Systems—SYBYL Software Method

**Figure 2:**
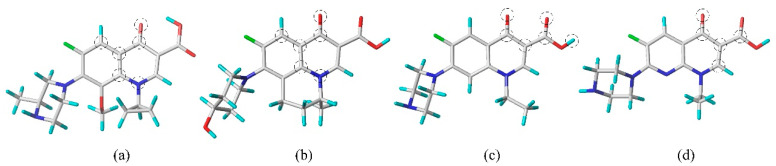
Template molecules of the 3D-QSAR model for the degradation capability of FQ molecules in advanced oxidation systems: (**a**) gatifloxacin; (**b**) nalidixic acid; (**c**) norfloxacin; (**d**) enoxacin. The atoms circled in the figure represent the common skeleton overlaid in the 3D-QSAR model (O is red, N is blue, F is green).

To delve into the degradation characteristics of FQ molecules in advanced oxidation systems, this study leveraged SYBYL-X2.0 software to build a 3D-QSAR model that predicts the degradation potential of FQ molecules. The study began by importing the structural files (mol2 format) of 16 commercially available FQ molecules into the “Compute—Minimize” module of SYBYL for conformational optimization, applying Gasteiger–Hückel charges to achieve the lowest possible energy state for the FQ structures (optimization parameters: Max Iterations: 10,000 Times [24]; Min Energy Change: 0.005 kcal/mol). The optimized FQ molecules were then randomly split into a training set and a test set at a ratio of about 3:1 [22]. The study constructed four 3D-QSAR models to represent the degradation capabilities of FQs for piperazine ring cleavage, defluorination, hydroxylation, and piperazine ring hydroxylation. Each model used a specific FQ molecule as a template, with a selection of FQ molecules assigned to the training and test sets (depicted in Figure 2). Finally, the “Align Database” module was utilized to superimpose the common skeletons based on the template molecules (Figure 2).

To explore the degradation characteristics of FQ molecules in advanced oxidation systems, a 3D-QSAR model was constructed using Comparative Molecular Similarity Index Analysis (CoMSIA). The model employed partial least squares (PLS) regression to establish a quantitative relationship between the three-dimensional structural features of FQ molecules and their degradation capabilities in advanced oxidation processes, followed by a validation of the model’s soundness [25]. The procedure involved the following steps: First, the leave-one-out (LOO) method was employed for cross-validation of the training set molecules to obtain the cross-validation coefficient q^2^ (greater than 0.5) and the optimal number of principal components N. Next, non-cross-validation regression analysis was performed to secure the non-cross-validation coefficient r^2^ (greater than 0.9), the standard error of estimation (SEE), and the test statistic F [26]. Finally, the test set underwent cross-validation to determine r^2^_pred_ (greater than 0.6). Once the model parameters were verified as reasonable, the 3D-QSAR model was completed [27]. Subsequently, the “3D-QSAR—View QSAR” function in SYBYL software was used to create three-dimensional contour maps of the CoMSIA model, which illustrate the degradation capabilities of FQ molecules in advanced oxidation systems (Figure S1). These contour maps provide a visual representation of the hydrophobic, electrostatic, steric, hydrogen bond acceptor, and hydrogen bond donor fields, facilitating an analysis of the interplay between the bond dissociation energy and molecular structure of FQ molecules in advanced oxidation processes.

### 2.4. Construction of Lasso Regression Model for the Degradation Capability of FQ Molecules in Advanced Oxidation Systems—Jupyter Notebook Tool

Machine learning (ML) is a sophisticated data mining technique that focuses on processing and distilling the interrelationships within complex datasets into mathematical representations [28]. The Lasso regression algorithm, known for constructing linear regression models with excellent stability and predictive precision [29], was selected for developing a 2D-QSAR model that predicts the bond dissociation energy of FQ molecules during advanced oxidation processes. The initial phase involved using the PaDEL 2.21-Descriptor software to forecast five principal structural property parameters across 11 commercially available FQ molecules featuring a piperazine ring structure. These parameters spanned geometric, electronic, physicochemical, spectral, and topological properties, with a comprehensive list of 1014 descriptors. In the subsequent phase, the decision tree algorithm was employed to prioritize these descriptors based on their impact on the FQ molecules’ bond dissociation energy. This prioritization process culminated in the identification of nine pivotal FQ molecular feature descriptors with the most significant correlation to bond dissociation energy. The final phase entailed the application of these nine descriptors as independent variables, with the bond dissociation energy of FQ molecules as the dependent variable, to compile and execute the Lasso regression algorithm. This predictive modeling endeavor served to further dissect and corroborate the correlation between the structural features of FQs and their degradation efficacy.

### 2.5. Toxicity Risk Assessment of Parent FQs and Their Degradation Products in Advanced Oxidation Systems—VEGA Software Method

In an effort to comprehensively assess the potential risks posed to human health and the environment by fluoroquinolone (FQ) antibiotics and their degradation products in advanced oxidation systems, this study utilized the VEGA 1.2.3 software to forecast a suite of 17 toxicological parameters. These indicators included the modulation of androgen and estrogen receptor activities, potential for carcinogenesis, chromosomal aberrations, mutagenicity, developmental toxicity, and screening for endocrine disruptor activities. The analysis further extended to hepatotoxicity, acute toxicity, ocular and dermal irritation, skin sensitization, chronic toxicity to aquatic algae, bioaccumulation potential as indicated by the bioconcentration factor, median lethal concentrations for Daphnia magna at 48 h (LC_50_DM), median lethal concentrations for fathead minnow at 96 h (LC_50_FM), and the degradation half-life (kM/Half-Life). The predictive outcomes were intended to furnish a nuanced assessment of the health and ecological risks associated with FQs and their oxidative degradation intermediates.

## 3. Results and Discussion

### 3.1. Evaluation of the Degradation Capability of FQ Molecules in Advanced Oxidation Systems Based on DFT Method

In the study delineated in Section 2.2, bond dissociation energies were quantified for 16 commercially available FQ molecules undergoing primary degradation in five distinct advanced oxidation processes, with the energy values being recalibrated into kJ/mol as presented in Table 1. This investigation sought to elucidate the relative degradation difficulty of various FQ molecules when subjected to advanced oxidation systems. An analysis of the average bond dissociation energies for 11 FQ molecules with a piperazine ring structure across five advanced oxidation processes revealed that LOM and OFL molecules possess the lowest bond energies, indicating these FQs are predisposed to facile degradation in advanced oxidation contexts. In contrast, molecules such as ENO, FLE, GAT, LEV, PEF, RUF, and SPA demonstrated the highest average bond energies, indicating reduced degradability within advanced oxidation processes. The CIP and NOR molecules had average bond energies that were intermediate, suggesting a certain level of degradability within advanced oxidation frameworks. Moreover, for a holistic comparison of the degradation ease of the 16 FQ molecules, this study also calculated the average bond dissociation energies for carbon–fluorine bonds in defluorination reactions, carbon–hydrogen bonds in hydroxylation reactions, and carbon–carbon bonds in decarboxylation reactions (as shown in Table 1). The findings suggested that molecules such as CIP, CLI, ENO, LEV, LOM, MOX, NOR, and OFL, with lower average energies for these three bond types, possess a higher degradation potential in advanced oxidation systems. Conversely, molecules like FLE, GAT, NAD, PEF, RUF, SPA, TOS, and GEM, with higher average bond energies, pose a greater challenge for degradation in advanced oxidation processes, necessitating focused attention on their degradability.

**Figure 3:**
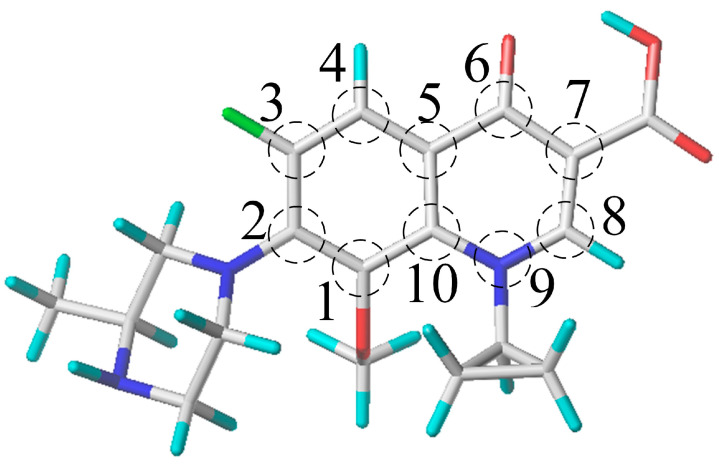
Basic skeleton site map of FQ molecules. (Numbers 1 to 10 represent position).

Structural analysis of different FQ molecules has indicated that in hydroxylation reactions, those with oxygenated heterocycles at the 1-position and 9-position (Figure 3) of the FQ framework (such as OFL and LEV) possess lower carbon–hydrogen bond energies. In contrast, molecules with sulfur-containing or carbon-only heterocycles at these positions (e.g., RUF, NAD) have higher carbon–hydrogen bond energies. In defluorination reactions, FQ molecules with an ethyl substituent at the 9-position (e.g., LOM, NOR, ENO, PEF) demonstrate lower carbon–fluorine bond energies than those with a cyclopropyl substituent (e.g., CLI, CIP, MOX, GEM, GAT, SPA). Yet, in piperazine ring cleavage reactions, the ethyl-substituted group shows higher carbon–nitrogen bond energies within the ring. Moreover, FQ molecules with a fluorine atom at the 1-position (e.g., LOM, FLE, SPA) have lower carbon–nitrogen bond energies in the piperazine ring than those lacking a fluorine atom (e.g., NOR), shedding light on why CIP, with a cyclopropyl group, has higher carbon–nitrogen bond energies in the piperazine ring than LOM with an ethyl group. Additionally, FQs with a methoxy group at the 1-position (e.g., GAT) have higher carbon–nitrogen bond energies in the piperazine ring, which may account for the high bond energies in GAT molecules despite the presence of a cyclopropyl group. Comparing NOR and PEF molecules, it was found that FQs with a para-methyl group on the piperazine ring (e.g., PEF) have not only lower carbon–nitrogen bond energies in the ring but also lower carbon–carbon bond energies in decarboxylation reactions. However, these molecules display higher bond energies for carbon–hydrogen, carbon–fluorine, and carbon–hydrogen in the piperazine ring during defluorination, hydroxylation, and piperazine ring hydroxylation reactions, respectively. This further explains the unusual higher carbon–fluorine bond energies in PEF molecules with an ethyl group compared to CIP with a cyclopropyl group.

Additionally, FQ molecules with an ethyl group at the 9-position have higher bond energies than those with a fluoroethyl group at the same position (FLE). However, the dual fluorine atoms connected to the benzene ring of the FLE molecule result in lower carbon–nitrogen bond energies in the piperazine ring compared to PEF. For SPA molecules, the elevated average bond energy is attributed to the amino group at the 4-position of the FQ core. The study also found that FQ molecules with a pyridine core (position 1 as nitrogen, ENO) have lower carbon–fluorine and carbon–carbon bond energies in defluorination and decarboxylation reactions, respectively, and higher carbon–hydrogen bond energies in hydroxylation reactions compared to those with a benzene core (position 1 as carbon, NOR). The carbon–nitrogen bond energies in the piperazine ring are lower, but the carbon–hydrogen bond energies on the ring are higher.

Collectively, it can be stated that FQ molecules with an ethyl substituent at the 9-position and a methoxy substituent at the 1-position of the fundamental skeleton exhibit a lower propensity for piperazine ring cleavage reactions. FQ molecules with a para-methyl group on the piperazine ring and a fluorine atom at position 1 of the core skeleton are more likely to experience such cleavage. Molecules with a cyclopropyl substituent at the 9-position and those with a pyridine moiety in the core are more susceptible to defluorination reactions. FQ molecules with an amino substituent at the 4-position and those with sulfur-containing or carbon-only heterocycles at the 1-position and 9-position are less likely to engage in hydroxylation reactions; in contrast, those with oxygen-containing heterocycles at these positions are more conducive to hydroxylation. Furthermore, FQs with an amino substituent at the 4-position and those with a pyridine moiety are less prone to undergo ring hydroxylation reactions.

### 3.2. Cluster-Analysis-Based Characterization of FQ Molecules’ Degradation Features in Advanced Oxidation Systems

**Figure 4:**
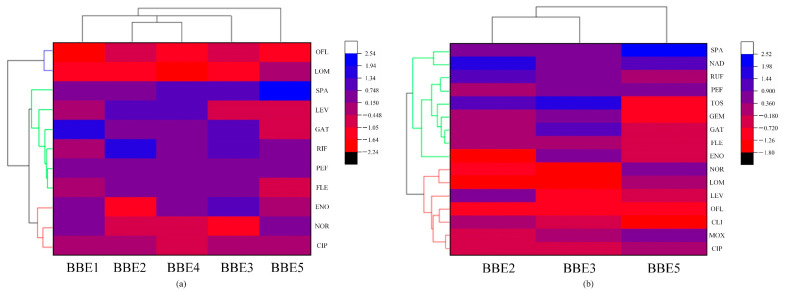
Cluster analysis graph of FQ molecules: (**a**) Cluster analysis of bond dissociation energies during the defluorination, decarboxylation, hydroxylation, piperazine ring breaking, and piperazine ring hydroxylation reactions of 11 FQ molecules. (**b**) Cluster analysis of bond dissociation energies during the defluorination, hydroxylation, and decarboxylation reactions of 16 FQ molecules.

Cluster analysis, an unsupervised machine learning technique, is adept at selecting representative features to streamline data dimensions and complexity, enabling a thorough investigation and visualization of the inherent relationships within data [30]. This approach is well suited for the current study’s need to deeply explore the link between the structural features of FQs and their degradation difficulty, despite the small data volume [30]. Using Origin software’s Heatmap tool, this study conducted cluster analysis on the bond dissociation energies of 11 FQ molecules across various reactions including defluorination, decarboxylation, hydroxylation, piperazine ring cleavage, and piperazine ring hydroxylation (Figure 4). The heatmap’s gradient from red to blue signifies increasing bond dissociation energies. The clustering outcome divides the FQ molecules into three distinct groups based on their degradation ease in advanced oxidation systems. LOM and OFL form one group, predominantly red on the heatmap, signifying their greater ease of degradation. FLE, GAT, LEV, PEF, RUF, and SPA are clustered into a second group, marked mostly by purple and blue, denoting a lower likelihood of degradation. ENO, CIP, and NOR constitute a third group, with a mix of red and purple, suggesting their degradation difficulty is intermediate between the two groups. Additionally, the row clustering of bond dissociation energies for defluorination, hydroxylation, and decarboxylation reactions categorizes the FQ molecules into two groups. NOR, LOM, LEV, OFL, CLI, MOX, and CIP, with a heatmap dominated by red, are more prone to degradation, while SPA, NAD, RUF, PEF, TOS, GEM, GAT, FLE, and ENO, with purple and blue, are less degradable. The overall analysis indicates that the cluster analysis results align well with the conclusions of Section 3.1.

To explore the collective tendencies in the ease of bond cleavage during the primary degradation reactions of FQ molecules in advanced oxidation systems, this study conducted a column cluster analysis of the bond dissociation energies for 16 FQ molecules across these five degradation processes (illustrated in Supplementary Figure S2, with missing values filled in using the average of the column). The analysis shows that the carbon–nitrogen bonds of FQ molecules have the lowest overall energies in piperazine ring cleavage and hydroxylation reactions, indicating a higher propensity for these reactions to occur in advanced oxidation systems. The carbon–fluorine bond energies are the highest in defluorination reactions, suggesting that defluorination is the most challenging reaction to take place. The carbon–hydrogen bond energies, including those in the piperazine ring, fall between the previous two categories, implying a moderate level of difficulty for hydroxylation and piperazine ring hydroxylation reactions within advanced oxidation systems.

### 3.3. Differential Analysis of the Degradation Capability of FQ Molecules in Advanced Oxidation Systems Based on 3D-QSAR Models

**Figure 5:**
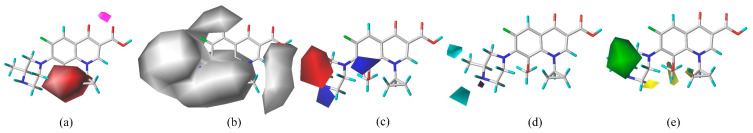
CoMSIA model contour maps of piperazine ring breaking in FQ molecules: (**a**) hydrogen bond acceptor field; (**b**) hydrophobic field; (**c**) electrostatic field; (**d**) hydrogen bond donor field; (**e**) steric field.

Utilizing bond dissociation energies from the primary degradation pathways of fluoroquinolone molecules, this research constructs a CoMSIA model to assess bond cleavage energies within advanced oxidation systems. It was found that the energy differences for decarboxylation among 16 commercial FQ molecules are below 2%, indicating that factors other than the core FQ structure have a minimal influence on this reaction. Attention is thus directed to CoMSIA models based on the bond dissociation energies from the other four degradation processes (evaluation parameters are shown in Table 2). The CoMSIA model for piperazine ring cleavage in FQ molecules, with a principal component of 5, exhibits a q^2^ value of 0.516, indicating robust predictive capabilities [31]. The model’s SEE of 0.001 and F-value of 227534.936, along with an R^2^ of 1.000, underscore its stability and predictive strength [31]. The external validation coefficient r^2^_pred_ of 0.972 and SEP of 0.043 further validate the model’s external predictive capacity [32]. All CoMISA models meet the evaluation criteria, attesting to the reliability of these models. The three-dimensional contour maps for the piperazine ring cleavage model (Figure 5 and Figure S2) demonstrate that the hydrogen bond acceptor, hydrophobic, and electrostatic fields collectively contribute 85.7% to the carbon–nitrogen bond, signifying their substantial influence on the cleavage reaction in FQ molecules. Detailed contour map information for the additional bond cleavage models is presented in Table 3.

The CoMSIA model’s contour maps for the piperazine ring cleavage of FQ molecules in advanced oxidation systems (Figure 5) show that the GAT molecule, in comparison to CIP, exhibits red blocks (indicative of the hydrogen bond acceptor field) and blue blocks (indicative of the electrostatic field) around the first position of its core structure. The bond energy of GAT’s piperazine ring carbon–nitrogen bond is 9.63% higher than that of CIP, suggesting increased resistance to cleavage for GAT in such oxidative environments. An analysis of the substituents at the 1-position of the core structures shows that GAT possesses a methoxy group, serving as both a hydrogen bond acceptor and an electronegative entity, unlike CIP, matching the contour map’s field patterns. Furthermore, the hydrophobic field contour map comparison between CIP and NOR shows white blocks around the 9-position of CIP’s core structure, and its piperazine ring’s carbon–nitrogen bond has a 5.83% lower bond energy than NOR’s, suggesting a greater ease of cleavage for CIP in advanced oxidation systems. The substituent analysis at the 9-position shows CIP’s cyclopropyl group to be more hydrophilic than NOR’s ethyl group, consistent with the hydrophobic field contour map.

In the advanced oxidation systems, the defluorination CoMSIA model’s hydrogen bond acceptor field contour map (Figure S2) for FQ molecules reveals magenta blocks surrounding the 1-position of the ENO molecule’s basic skeleton. The carbon–fluorine bond energy in ENO is 0.82% lower than that in NOR, suggesting an increased likelihood of defluorination for ENO. An analysis of the substituents at the first position of the core structures of these FQ molecules shows that ENO has a nitrogen atom that serves as a hydrogen bond acceptor, unlike NOR. This is in agreement with the hydrogen bond acceptor field observed in the contour maps. Furthermore, the hydrophobic field contour map comparison between GAT and LOM reveals yellow blocks around the 1-position of LOM’s core structure, with LOM’s carbon–fluorine bond energy being 2.05% lower than GAT’s, suggesting LOM is more susceptible to defluorination. The substituent analysis at the 1-position shows that LOM’s ethyl group is more hydrophobic than GAT’s cyclopropyl group, consistent with the hydrophobic field contour map.

The hydrophobic field contour map for the hydroxylation CoMSIA model of FQ molecules in advanced oxidation systems (Figure S2) shows that SPA molecules exhibit yellow blocks at the 4-position of their basic skeleton, in contrast to LOM molecules. The carbon–hydrogen bond energy in SPA is 3.31% higher than that in LOM, suggesting a decreased likelihood of hydroxylation for SPA. Further comparison of the characteristics of the groups at the 4-position of the basic skeleton of the two aforementioned FQ molecules reveals that the SPA molecule possesses a more hydrophilic amino group, while the one at the 4-position of the LOM molecule possesses a more hydrophobic H atom, which coincides with the color-blocking pattern of the hydrophobic field in the contour maps. In the piperazine ring hydroxylation CoMSIA model for FQ molecules (Figure S2), SPA exhibits magenta blocks at the fourth position of its basic skeleton when compared to LOM, and the carbon–hydrogen bond energy in SPA is 7.27% higher than that in LOM, indicating a greater resistance to piperazine ring hydroxylation. The presence of an amino group in SPA, acting as a hydrogen bond donor, compared to the lack of such a group in LOM, matches the hydrophobic field patterns observed in the contour maps.

The analysis leads to the conclusion that FQ molecules with either a hydrogen bond donor or a positive group at the 1-position and a hydrophilic group at the 9-position of their basic skeleton have an increased likelihood of carbon–nitrogen bond cleavage within the piperazine ring. Molecules with a hydrogen bond donor or hydrophobic group at the 1-position are more inclined to participate in defluorination reactions. Those with hydrophobic groups at the 4-position are more amenable to hydroxylation reactions. FQ molecules with a hydrogen bond acceptor group at the 4-position of their basic skeleton are more readily subject to piperazine ring hydroxylation.

### 3.4. Toxicity Risk Assessment of Degradation Products of FQ Molecules in Advanced Oxidation Systems Based on Toxicokinetic Models

Although advanced oxidation processes demonstrate significant efficacy in the removal of FQ molecules, their constrained mineralization ability results in the formation of numerous intermediates with potential toxicity, leading to ecological concerns [1]. Studies have identified that out of 30 potential degradation products of ciprofloxacin, 28 pose risks of mutagenicity and are challenging to biodegrade, with all products carrying certain levels of toxicity and carcinogenicity [33]. Likewise, the 25 degradation products of norfloxacin are characterized by mutagenicity, toxicity, carcinogenicity, and biodegradation resistance [33]. The objective of this study is to investigate the toxicity variation of intermediate products during the degradation of 16 commercial FQ molecules, aiming to circumvent pathways with toxic intermediates and reduce the potential toxicity risks associated with FQs and their degradation products. The research employs VEGA 1.2.3 software for the risk assessment of human health and the ecological impact of these FQ molecules and their intermediates (Table S1).

#### 3.4.1. Human Health Risk Assessment of Degradation Products of FQ Molecules in Advanced Oxidation Systems

Table S1 indicates that the 16 commercial FQ molecules are assessed as inactive for both estrogenic and androgenic receptor-mediated effects, as well as for endocrine-disrupting activity, suggesting no associated risk from these FQ molecules in these areas. Further risk analysis for carcinogenicity, mutagenicity, and chromosomal aberrations among the 16 FQ molecules reveals that, apart from LOM and MOX, the rest exhibit potential risks in one or more of these categories. PEF and GEM, in particular, are identified as having all three potential genotoxic “triad” effects, corroborating findings from research by Segalin et al. [33]. Evaluations of developmental toxicity and hepatotoxicity risks show that all 16 FQ molecules are implicated, with 12 of them—CIP, CLI, ENO, FLE, GAT, MOX, NAD, NOR, PEF, RUF, SPA, and TOS—facing risks in both categories. Risk assessments for eye irritation, skin sensitization, and skin irritation among the 16 commercial FQ molecules reveal risks of eye irritation or skin sensitization for all molecules, with no skin irritation risks identified. The acute toxicity assessment, based on LD50 predictions, reveals significant variability, identifying RUF as the molecule with the highest acute toxicity risk and ENO as the least risky. Furthermore, the study employs a negative index standardization approach to process acute toxicity data, calculating a comprehensive human health risk index for each FQ molecule, with PEF presenting the highest risk and LOM the lowest.

An analysis of Table S1 for the 16 commercial FQ molecules and their degradation products indicates that the risk assessments for estrogenic and androgenic receptor-mediated effects, as well as endocrine-disrupting activities, are consistently inactive. This demonstrates that the degradation intermediates of the 16 FQ molecules do not exhibit a significant change in risk levels for these effects and activities compared to the parent FQ molecules. Further scrutiny reveals that, except for MOX, the degradation intermediates of the other 15 FQ molecules all exhibit potential carcinogenic, mutagenic, or chromosomal aberration risks. To explore the trends of these risks during degradation, the study assigns a value of 0 for no risk and 1 for risk presence, calculating a composite index for the “triad” risks of the FQ molecules and their intermediates (Table S1). The results indicate that the degradation intermediates of CIP, CLI, ENO, FLE, LEV, LOM, NAD, RUF, and SPA have an increased average “triad” risk index compared to their parent molecules, suggesting an overall elevation in these risks during the degradation process. The developmental toxicity and hepatotoxicity risk assessments indicate that all intermediates of the 16 FQ molecules carry these risks, with the exception of GEM and some intermediates of FLE, all other intermediates exhibit both risks. The composite index for these risks (Table S1) shows that the degradation intermediates of LEV, LOM, and OFL have increased developmental toxicity and hepatotoxicity risks compared to the parent molecules, while the risks for the intermediates of other FQ molecules remain the same or decrease. Moreover, all FQ molecule degradation intermediates, except for some intermediates of FLE, GAT, and LEV, present risks of eye irritation, skin sensitization, or skin irritation. Notably, only two intermediates of SPA do not pose a risk of skin irritation. The composite index for these risks (Table S1) reveals that the degradation intermediates of SPA, RUF, and GEM show an increasing trend in these risks compared to the parent molecules, while the risk levels for the intermediates of NAD and TOS remain unchanged, and the risks for the intermediates of the other 11 FQ molecules decrease. The acute toxicity risk assessment results indicate that, apart from LEV, OFL, RUF, and TOS, the acute toxicity of the degradation intermediates of the other FQ molecules generally shows an upward trend (e.g., the primary degradation products of ENO show an average increase in toxicity of 25.42% over the parent molecule). In addition, the overall human health risk index for the degradation intermediates of the 16 FQ molecules shows that, except for LEV, LOM, NAD, RUF, SPA, and GEM, the human health risks for the remaining 10 FQ molecules decrease in varying degrees during degradation.

To assess the human health risks of intermediates from various FQ degradation pathways, this study adds up the overall human health risk indices of intermediates from the same pathway and computes their average (Table S1, excluding FQ molecules with missing data). The findings indicate that the overall human health risk index for degradation products from pathway P1 is reduced by 11.13%, which is the largest reduction among all primary degradation pathways. As advanced oxidation systems continue to break down primary degradation products, there is a trend of increasing human health risks for later-stage degradation products, with the overall risk index for tertiary degradation products surpassing the parent molecules by 0.48%. Moreover, the overall human health risk indices for primary degradation products from pathways P3 and P4 are lower than the parent molecules, but the secondary degradation products from pathway P4 show a higher risk than the parent molecules (an increase of 2.19% in the overall index). Compared to the parent molecules, degradation products from pathways P2 and P5 show increased human health risks (with overall indices rising by 3.69% and 4.08%, respectively). A detailed analysis of the degradation products with increased human health risks compared to the parent molecules during FQ degradation shows that these products consistently contain hydroxyl groups, suggesting that hydroxyl groups may enhance the potential human health risks of the degradation products. In summary, the use of parent molecules with substantial human health risks (like PEF) and molecules whose risks increase during the advanced oxidation degradation process (such as LEV, LOM, NAD, RUF, SPA, and GEM) should be minimized to mitigate potential health hazards.

#### 3.4.2. Ecological Environmental Risk Assessment of Degradation Products of FQ Molecules in Advanced Oxidation Systems

To conduct a holistic evaluation of the ecological and environmental risks posed by FQ molecules and their degradation intermediates, this study integrates five key assessment indicators: chronic toxicity to algae, 48 h LC_50_ for Daphnia magna, 96 h LC50 for Pimephales promelas, bioconcentration factor, and half-life. Since lower values for the first three indicators correlate with higher ecological risks, these are treated as negative indicators. Conversely, larger values for bioconcentration factor and half-life indicate higher ecological risk, so these are treated as positive indicators. The bio-toxicity risk index is derived by summing the processed values for the first three indicators, and the total ecological and environmental risk index is calculated by summing the processed values for all five indicators, as detailed in Table S1. Upon analysis, it is found that of the 16 parent FQ molecules, TOS exhibits the highest bio-toxicity risk, while ENO presents the lowest. The bioconcentration factor assessment indicates that TOS has the highest potential for bioaccumulation, whereas LEV, NOR, and OFL have the lowest. The half-life assessment shows that MOX has the longest half-life, and FLE has the shortest. The comprehensive ecological and environmental risk index suggests that TOS carries the highest risk, while ENO carries the lowest.

A comparative analysis of the ecological and environmental risks of the 16 parent FQ molecules and their degradation products indicates that, with the exception of CLI, MOX, and SPA, the bio-toxicity risk index for the intermediates of the remaining FQ molecules decreased during degradation when compared to the parent molecules (for example, the primary degradation products of ENO showed a 22.67% reduction in average bio-toxicity compared to the parent molecule). In terms of bioaccumulation, except for the intermediates of CIP, which exhibited no significant change, the overall bioaccumulation potential of the degradation products of the other 15 FQ molecules decreased (for instance, the primary degradation products of CLI showed a 22.73% reduction in average bioaccumulation potential compared to the parent molecule). The half-life assessment indicated that, except for NAD, which had an increased half-life, the half-lives of the other 15 FQ molecules were reduced during degradation (for example, the primary degradation products of ENO showed a 39.44% reduction in average half-life compared to the parent molecule). The comprehensive ecological and environmental risk index showed a decrease for all 16 FQ molecules during degradation (for instance, the overall ecological and environmental risk index for the primary degradation products of PEF decreased by 16.76% compared to the parent molecule). Overall, the production or widespread use of molecules with the highest ecological and environmental risks (such as TOS), those with the longest half-lives (such as MOX), those with increased combined toxicity during degradation (such as CLI, MOX, and SPA), and those with extended half-lives (such as NAD) should be avoided. Additionally, to investigate the ecological and environmental risks of intermediates from different FQ degradation pathways, this study aggregates the overall ecological and environmental risk indices of intermediates from the same pathway and calculates their average. The results indicate that the primary degradation products from all five pathways pose a lower ecological and environmental risk than before degradation, with pathway P2 yielding the lowest risk (a 16.58% decrease in the overall index). In pathways P1, P4, and P5, the ecological and environmental risks of intermediates further decrease as degradation progresses.

Upon comprehensive evaluation, it is evident that the parent molecules of PEF, TOS, and MOX present significant risks to human health or the ecological environment. The degradation intermediates of CLI, MOX, LEV, LOM, NAD, RUF, SPA, and GEM exhibit increased risks to human health or the ecological environment compared to their respective parent molecules. Consequently, the production or extensive use of these compounds should be avoided. Furthermore, degradation products resulting from the piperazine ring cleavage reaction have the lowest aggregate index for human health risk, while those from defluorination and decarboxylation reactions have the highest. Therefore, it is recommended to minimize the occurrence of these two reactions during advanced oxidation degradation processes.

### 3.5. Mechanistic Analysis of the Differential Degradation Capability of FQ Molecules in Advanced Oxidation Systems

#### 3.5.1. Differential Analysis of the Degradation Capability of FQ Molecules in Advanced Oxidation Systems Based on 2D-QSAR Model

To delve deeper into the causes of the variability in the degradation capabilities of FQ molecules, this study selected 11 FQ molecules with piperazine rings to construct Lasso regression models for defluorination, hydroxylation, piperazine ring cleavage, and piperazine ring hydroxylation reactions. Initially, a comprehensive set of molecular descriptors was calculated for the 11 molecules, including geometric, electronic, physicochemical, spectral, and topological parameters (a total of 1014 feature parameters), to establish a molecular feature database for the Lasso regression models. Previous research has indicated that a larger number of relevant feature parameters can enhance the reliability of model predictions to some extent, but may also lead to low training efficiency or excessive prediction bias [34]. Therefore, this study employed a decision tree method to identify the importance of the 1014 feature parameters for the 11 commercial FQ surrogate molecules. The top nine feature parameters, prioritized by importance, served as independent variables, and the bond-breaking energy (BBE) during the advanced oxidation degradation processes of the FQs served as the dependent variable. The datasets were split into training and testing sets in a 7:3 ratio to construct Lasso regression models for piperazine ring cleavage, defluorination, hydroxylation, and piperazine ring hydroxylation reactions (Table 4). The results showed that the Lasso regression model for piperazine ring cleavage had an R^2^ of 0.731, the defluorination model had an R^2^ of 0.770, the hydroxylation model had an R^2^ of 0.885, and the piperazine ring hydroxylation model had an R^2^ of 0.825. These findings demonstrate that the Lasso regression models for the advanced oxidation degradation of the 11 commercial FQ surrogate molecules meet the requirements for significance testing and possess good predictive capabilities [35]. The model equations are as follows:

(2) $$\begin{array}{c} \mathrm{BBE}1=20.785{\left(\mathrm{ALogP}\right)}^{2}+39.707\mathrm{MATS}2m-98.611\mathrm{maxsssCH}+6.559\mathrm{GATS}5c\\ +0.530\mathrm{ATS}6s+1878.111\mathrm{MATS}1m-50.654\mathrm{SP}-2-48.891\mathrm{SM}1\_\mathrm{Dze}\\ +275.920\mathrm{AVP}-1+568.480 \end{array}$$

(3) $$\begin{array}{c} BBE2=0.137ECCEN-66.598MATS8i-62.187MATS3c+1.367nHaaCH\\ -19.339SpMin3_{\mathrm{Bhm}}-5.192SpMin8_{\mathrm{Bhi}}+0.136{\mathrm{EE}}_{\mathrm{Dzp}}+3.848MAXDP\\ +397.123 \end{array}$$

(4) $$\begin{array}{c} BBE3=7.067{\left(\mathrm{ALogP}\right)}^{2}+48.210GATS6e+1.091SP-2+21.735GATS3i\\ -1.089SpMAD\_Dt-24.991GATS5i+4.360C1SP2+368.843 \end{array}$$

(5) $$\begin{array}{c} BBE4=7.224MLFER\_BH+5.962SP-2-183.529GATS6i+0.525Sare\\ -5.857SpMax8\_Bhe+10.888SP-5-24.674GATS6e+0.004ATS0m\\ -0.003ATS3i+620.923 \end{array}$$

As illustrated in Equation (2), the nine most significant feature parameters in the Lasso regression model for piperazine ring cleavage include ALOGP descriptors (ALogP)^2^, autocorrelation descriptors (MATS2m, GATS5C, ATS6s, MATS1m), atomic-type electrotopological state descriptors (maxsssCH), Chi path descriptors (SP-2, AVP-1), and matrix-based descriptors (SM1_Dze). The positive coefficients associated with (ALogP)^2^, GATS5C, ATS6s, MATS2m, and AVP-1 demonstrate a positive correlation with the bond energy of the carbon–nitrogen bonds in the piperazine rings of FQ molecules, indicating that an increase in these feature parameters correlates with an increase in bond energy. Conversely, the coefficients for MATS1m, maxsssCH, SP-2, and SM1_Dze are negative, suggesting an inverse relationship with the bond energy of the carbon–nitrogen bonds in the piperazine rings, meaning that as the values of these feature parameters increase, the bond energy tends to decrease. Among these, the feature importance of (ALogP)^2^ is the highest (0.713) compared to the other eight parameters. ALogP represents a parameter for molecular lipophilicity, with higher values indicating more pronounced lipophilic properties of the molecule [36]. The analysis suggests a significant positive correlation between the bond energy of the carbon–nitrogen bonds in the FQ molecules’ piperazine rings and the lipophilicity of the FQ molecules. That is, the higher the ALogP value of an FQ molecule, the greater the bond energy of the carbon–nitrogen bonds in its piperazine ring, making the piperazine ring cleavage reaction less likely to occur in AOPs. This finding is consistent with research by Liu et al., who observed that antibiotics with higher hydrophobicity were more susceptible to degradation through activated carbon ultrasonic degradation [37].

Figure S2 illustrates that the presence of white coloration around the ninth position of the FQ molecule signifies that FQ molecules with hydrophilic groups at this position are more prone to piperazine ring carbon–nitrogen bond cleavage. This observation allows for the Lasso regression model for piperazine ring cleavage in advanced oxidation systems to be corroborated by the CoMSIA model for the same reaction. As shown in Equation (3), in the defluorination Lasso regression model, the eight most significant feature parameters include the eccentric connectivity index descriptor (ECCEN), autocorrelation descriptors (MATS8i, MATS3c), electrotopological state atomic descriptors (nHaaCH, MAXDP), charge-modified eigenvalue descriptors (SpMin3_Bhm, SpMin8_Bhi), and the Barysz matrix descriptor (EE_Dzp). The positive coefficients for ECCEN, nHaaCH, EE_Dzp, and MAXDP indicate a direct correlation with the bond energy of the carbon–fluorine bonds in FQ molecules, meaning that as these feature parameters increase, the bond energy of the carbon–fluorine bonds tends to strengthen. Conversely, the negative coefficients for MATS8i, MATS3c, SpMin3_Bhm, and SpMin8_Bhi suggest an inverse relationship, indicating that as these feature parameters increase, the bond energy of the carbon–fluorine bonds tends to decrease. Among these, ECCEN has the highest feature importance (0.737) compared to the other seven parameters. ECCEN is a topological descriptor that characterizes the topological properties of a chemical molecule by combining information about the distances and adjacency of atoms within the molecule [38]. Specifically, the ECCEN descriptor calculates the eccentricity (the maximum distance from an atom to all other atoms in the molecule) for each atom, multiplies it by the atom’s connectivity (number of adjacent atoms), and then sums these values to obtain the ECCEN value for the entire molecule. This indicates that the bond energy of the carbon–fluorine bonds in FQ molecules is significantly positively correlated with the overall eccentricity of the molecule, meaning that the greater the overall eccentricity of an FQ molecule, the higher the bond energy of its carbon–fluorine bonds. FQ molecules with lower ECCEN values (CIP, ENO, LOM, NOR) tend to have a simpler basic skeleton and fewer substituents on the piperazine ring, correlating with lower carbon–fluorine bond energy. Therefore, FQ molecules with fewer substituents and simpler structures are more likely to undergo defluorination reactions in advanced oxidation systems. Tian et al. studied the degradation of recalcitrant organic compounds such as humic acids using a combined process (UASB-A/O-USSB) and found that organic compounds with simple molecular structures are preferentially degraded through anaerobic digestion and short-range denitrification [39]. Zhu et al. investigated the degradation of organic dyes using magnetic catalysts and found that smaller and simpler molecular structures are more easily degraded [40].

As can be seen from Equation (4), within the hydroxylation Lasso regression model, the seven most significant feature parameters include ALOGP descriptors (ALogP)^2^, autocorrelation descriptors (GATS6e, GATS3i, GATS5i), Chi path descriptors (SP-2), Detour matrix descriptors (SpMAD_Dt), and carbon-type descriptors (C1SP2). The coefficients for (ALogP)^2^, GATS6e, SP-2, GATS3i, and C1SP2 are positive, indicating a positive correlation with the bond energy of the carbon–hydrogen bonds in FQ molecules, suggesting that as these feature parameters increase, the bond energy of the carbon–hydrogen bonds tends to strengthen. Conversely, the negative coefficients for SpMAD_Dt and GATS5i imply an inverse relationship, indicating that as these feature parameters increase, the bond energy of the carbon–hydrogen bonds tends to decrease. Among these, (ALogP)^2^ has the highest feature importance (0.847), signifying a significant positive correlation between the bond energy of the carbon–hydrogen bonds in FQ molecules and the lipophilicity of the FQ molecules; that is, the higher the ALogP value of an FQ molecule, the greater the bond energy of its carbon–hydrogen bonds. As shown in Figure S2, the presence of white patches around the 1-position of the FQ molecule indicates that FQ molecules with hydrophilic groups at this site have carbon–hydrogen bonds that are more susceptible to cleavage. This finding allows for the hydroxylation Lasso regression model for FQ molecules in advanced oxidation systems to be corroborated by the CoMSIA model for the hydroxylation of FQ molecules in the same systems.

As demonstrated in Equation (5), within the Lasso regression model for hydroxylation of the piperazine ring, the nine most significant feature parameters include MLFER descriptors (MLFER_BH), Chi path descriptors (SP-2, SP-5), autocorrelation descriptors (GATS6i, GATS6e, ATS0m, ATS3i), constitutional descriptors (Sare), and charge-modified eigenvalue descriptors (SpMan8_Bhe). The coefficients for MLFER_BH, SP-2, Sare, SP-5, and ATS0m are positive, indicating a positive correlation with the bond energy of the carbon–hydrogen bonds in the piperazine rings of FQ molecules, indicating that as these feature parameters increase, the bond energy of the carbon–hydrogen bonds tends to strengthen. In contrast, the negative coefficients for GATS6i, SpMan8_Bhe, GATS6e, and ATS3i suggest a negative correlation, indicating that as these feature parameters increase, the bond energy of the carbon–hydrogen bonds tends to decrease. Among these, MLFER_BH, which is a descriptor that characterizes the hydrogen bond basicity of a molecule, has the highest feature importance (0.749). A higher MLFER_BH value indicates a stronger ability of the molecule to act as a hydrogen bond acceptor and form intermolecular hydrogen bonds with other hydrogen bond donors [41]. This finding is consistent with a significant positive correlation between the bond energy of the carbon–hydrogen bonds in the piperazine rings of FQ molecules and the molecules’ ability to form hydrogen bonds as acceptors; the stronger the ability of an FQ molecule to form hydrogen bonds as an acceptor, the higher the bond energy of the carbon–hydrogen bonds in its piperazine ring. Figure S2 shows purple patches around the 4-position and 8-position of the FQ molecule, indicating that FQ molecules with hydrogen bond donor groups at these positions have piperazine ring carbon–hydrogen bonds that are more susceptible to cleavage. This indicates that the Lasso regression model for piperazine ring hydroxylation in advanced oxidation systems can be validated against the CoMSIA model for the hydroxylation of the piperazine ring in advanced oxidation systems.

In the realm of advanced oxidation systems, this research has elucidated several key insights regarding FQ molecules. Firstly, FQ molecules that possess hydrophilic groups at the 9-position exhibit a heightened susceptibility to the cleavage of carbon–nitrogen bonds within the piperazine ring. Secondly, FQ molecules characterized by a paucity of substituents and a simplistic structure are more likely to undergo defluorination reactions. Thirdly, the presence of hydrophilic groups at the 1-position of FQ molecules correlates with an increased propensity for carbon–hydrogen bond cleavage. Fourthly, FQ molecules that feature hydrogen bond donor groups at the 4-position and 8-position are more prone to the disruption of carbon–hydrogen bonds in the piperazine ring. The analytical conclusions derived from the Lasso regression models for chemical bond cleavage in FQ molecules are in concordance with the findings from 3D-QSAR models, indicating a robust consistency between these modeling approaches.

#### 3.5.2. Mechanistic Analysis of the Differential Degradation Capability of FQ Molecules in Advanced Oxidation Systems Based on Fukui Function

**Figure 6:**
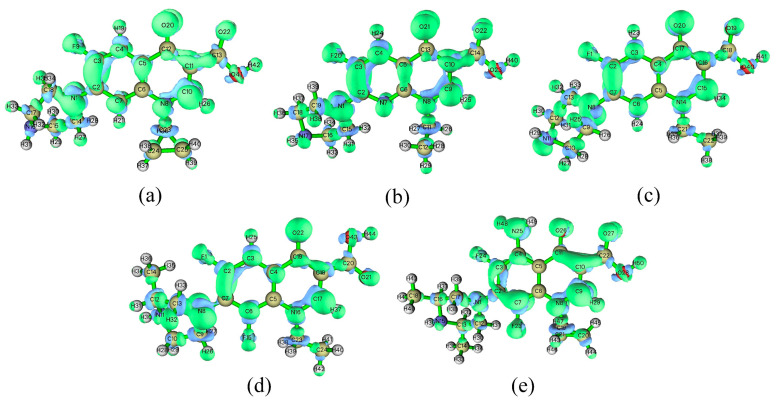
Fukui function isosurface maps of FQ molecules: (**a**) CIP; (**b**) ENO; (**c**) NOR; (**d**) LOM; (**e**) SPA.

The Fukui function is a commonly used real-space function for predicting potential reactive sites within molecules, extensively applied across the fields of biology, medicine, chemistry, and environmental science. Predictive outcomes are typically categorized into three types: nucleophilic attack (f^+^), electrophilic attack (f^−^), and radical attack (f^0^), with higher predictive values indicating stronger reactivity of atoms within the molecule [42]. In this study, the Fukui function is employed to assess the relative ease of degradation of different FQ molecules within advanced oxidation systems, thereby investigating the mechanistic differences in the advanced oxidation degradation capabilities of FQ molecules (Table S2). To investigate the mechanistic impact of specific functional groups on molecular degradation capabilities based on the Fukui function, this study selected two molecules with highly similar core structures for comparative analysis. These molecules are NOR and CIP, which differ in their substituents at the 9-position; ENO and NOR, which have different atoms at the first position; and LOM and SPA, which vary in the substituents at the 4-position (Figure 6).

An analysis of the Fukui function predictive values for the NOR molecule reveals that atoms such as O20, C15, C3, and N8 are most susceptible to radical attack. Bao et al. have also confirmed through the Fukui function and electrostatic potential maps that the piperazine ring is more prone to radical attack relative to other parts of the NOR molecule [15,43]. For the CIP molecule, atoms such as O20, N1, C3, and C10 are most affected by radicals, indicating these atoms are more likely to be attacked by radicals in advanced oxidation processes. Notably, the N8 atom in the NOR molecule and the N1 atom in the CIP molecule correspond to the nitrogen atoms on their respective piperazine rings. A comparison of their f^0^ values shows that the f^0^ value for the N8 atom in NOR is 18.37% higher than that for the N1 atom in CIP, suggesting that the nitrogen atom on the piperazine ring of CIP is more susceptible to radical attack compared to that of NOR. Additionally, the bond energy of the carbon–nitrogen bond in the piperazine ring of CIP is 5.83% lower than that of NOR, indicating that a higher f^0^ value for the nitrogen atom may increase the likelihood of piperazine ring cleavage in advanced oxidation systems. Furthermore, a comparison of the molecular structural features of CIP and NOR reveals that the substituent at the 9-position in CIP is a cyclopropyl group, while in NOR, it is an ethyl group. This suggests that compared to the ethyl group, the cyclopropyl group at this position of FQ molecules may enhance the reactivity of the nitrogen atom on the piperazine ring, thereby making the ring more susceptible to cleavage.

An analysis of the Fukui function predictive values for the ENO molecule indicates that atoms such as O21, C3, C4, C9, and N1 are most susceptible to radical attack. Ao et al. also utilized the Fukui function to determine that the carbon atoms connected to the fluorine atoms in the LEV molecule are relatively prone to radical attacks [15]. The C3 atom in ENO and the C2 atom in NOR are the carbon atoms in the basic FQ skeleton connected to fluorine atoms. A comparison of their f0 values reveals that the f^0^ value for the C3 atom in ENO is 26.28% higher than that for the C2 atom in NOR, suggesting that the fluorine atom on the basic skeleton of ENO is more susceptible to radical attack compared to that of NOR. Moreover, the bond energy of the carbon–fluorine bond in ENO is 0.82% lower than that in NOR, indicating that the higher the f^0^ value of the carbon atom connected to the fluorine atom in FQ molecules, the greater the likelihood of defluorination reactions occurring in advanced oxidation systems. Further structural analysis of ENO and NOR reveals that the basic skeleton of ENO contains a pyridine ring (with the nitrogen atom at the 1-position), while the basic skeleton of NOR contains a benzene ring (with the carbon atom at the 1-position). This suggests that, compared to a benzene ring structure, a pyridine structure on the basic skeleton of FQ molecules enhances the radical reactivity of the carbon atom connected to the fluorine atom, thereby increasing the propensity for defluorination in FQ molecules.

The Fukui function predictive values for the LOM molecule indicate that atoms such as N8, C17, and O22 are most susceptible to radical attack, while in the SPA molecule, atoms like C7, C9, N25, O26, and O27 are most prone to radical attack. Notably, the C13 atom in LOM and the C17 atom in SPA are carbon atoms on their respective piperazine rings. A comparison of the f^0^ values for C13 and C17 reveals that the f^0^ value for the C13 atom in LOM is 130.61% higher than that for the C17 atom in SPA, suggesting that the carbon atom on the piperazine ring of LOM is more readily attacked by radicals compared to that of SPA. Additionally, the carbon–hydrogen bond energy in LOM’s piperazine ring is 7.27% lower than that in SPA, suggesting that a higher f^0^ value for carbon atoms increases the likelihood of piperazine ring hydroxylation in FQ molecules. Further analysis of the substituent characteristics of LOM and SPA reveals that the 4-position in SPA is occupied by an amino group, while in LOM, it is a hydrogen atom. This suggests that, compared to an amino group, a hydrogen atom at the 4-position of FQ molecules may enhance the reactivity of carbon atoms in the piperazine ring, leading to a higher likelihood of hydroxylation reactions on the piperazine ring.

In summary, this study finds that in the advanced oxidation process of FQ molecules, atoms with higher f^0^ values facilitate the cleavage reactions of chemical bonds associated with those atoms. Moreover, adding a cyclopropyl group at the 9-position of FQ molecules, replacing the benzene ring structure on the basic skeleton with a pyridine structure, and avoiding the addition of an amino group at the 4-position are conducive to enhancing the reactivity of atoms involved in advanced oxidation reactions. Consequently, these modifications increase the propensity of FQ molecules to undergo piperazine ring cleavage, defluorination, and hydroxylation.

#### 3.5.3. Mechanistic Analysis of the Differential Degradation Capability of FQ Molecules in Advanced Oxidation Systems Based on Fukui Function

**Figure 7:**
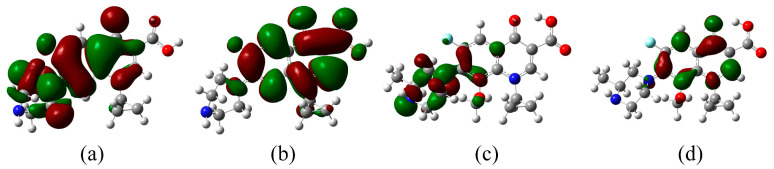
HOMO and LUMO distribution maps of FQ molecules: (**a**) HOMO distribution map of CIP molecule; (**b**) LUMO distribution map of CIP molecule; (**c**) HOMO distribution map of GAT molecule; (**d**) LUMO distribution map of GAT molecule.

In an effort to decode the reactivity trends of FQ molecules with radicals within advanced oxidation systems, this research focused on CIP and GAT molecules, which, while structurally similar, differ in the substituents at the 1-position. The Multiwfn 3.8 software [44] was employed to calculate the highest occupied molecular orbital (HOMO) and the lowest unoccupied molecular orbital (LUMO) energies of 16 optimized FQ molecules (Figure 7), analyzing their reactivity with radicals. It was found that the strongest reactivity between two reactants occurs when they exhibit the most favorable interaction energy, a phenomenon closely associated with the frontier molecular orbitals of the interacting substances [45]. Therefore, considering the HOMO-LUMO energy gap (ΔE) of FQ molecules and radicals is crucial, as a smaller energy gap indicates stronger interactions between the orbitals [45]. The hydroxyl radical, due to its prevalent and highly reactive nature in advanced oxidation systems, was selected to probe the reactivity of FQ molecules. The LUMO energy value of the optimized hydroxyl radical structure was calculated using the Multiwfn 3.8 software.

An analysis of the HOMO and LUMO distributions of CIP and GAT molecules reveals that the HOMOs of CIP are primarily located on the two ring structures of its core skeleton (atoms C2, C3, C5, C6, C11, C10, N8, etc.), the piperazine ring (atoms N1, C18, C14, C17, C15, etc.), and the atoms F9 and O20, indicating that these structural parts of the CIP molecule have a strong electron-donating capability. The LUMOs are mainly distributed over the two ring structures of the core skeleton (atoms C2, C3, C4, C6, C7, N8, C10, C11, C12, etc.), the piperazine ring (atom N1), the carboxyl group (atoms C13, O22, O41), and the atoms F9 and O20, suggesting that these structural parts of the CIP molecule have a strong electron-accepting capability. For GAT, the HOMOs are mainly located on the two ring structures of its core skeleton (atoms C2, C3, C4, C6, etc.), the piperazine ring (atoms N8, C13, C9, N11, etc.), the methoxy group (atom O14), and the atoms F1 and O22, indicating that these structural parts of the GAT molecule have a strong electron-donating capability. The LUMOs are mainly distributed over the two ring structures of the core skeleton (atoms C2, C3, C4, C6, C7, N16, C17, C19, etc.), the piperazine ring (atom N8), the methoxy group (atom O14), and the atom O22, suggesting that these structural parts of the GAT molecule have a strong electron-accepting capability. This is in line with the analysis by Ao et al. on the LEV molecule, which found a rich distribution of HOMOs near the piperazine ring and core skeleton, and LUMOs concentrated around the core skeleton and carboxyl group. Further comparison of the HOMO distribution maps of CIP and GAT molecules shows that the HOMOs of CIP are more densely distributed over the piperazine ring, the carbon atoms involved in defluorination, and the carbon atoms involved in hydroxylation reactions than those of GAT, indicating that in advanced oxidation systems, CIP is more likely than GAT to undergo piperazine ring cleavage, defluorination, hydroxylation, and hydroxylation of the piperazine ring.

Furthermore, a comparison of the ΔE between CIP and GAT molecules reveals that the ΔE of CIP is 5.19% lower than that of GAT, indicating stronger interactions between the orbitals of CIP and the hydroxyl radical and thus a higher reactivity of CIP with the hydroxyl radical compared to GAT. Asghar et al. found that the interaction between Acid Blue dye and ozone was the strongest by calculating the HOMO of the dye and the LUMO of various oxidizing agents [45]. A structural comparison between CIP and GAT molecules shows that the 1-position of GAT is occupied by a methoxy group, while in CIP, it is a hydrogen atom. This suggests that the addition of a methoxy group at the 1-position of FQ molecules can cause the highest occupied molecular orbital to be further removed from the carbon atoms involved in the piperazine ring, defluorination, and hydroxylation reactions, leading to an increase in the ΔE between FQ molecules and the hydroxyl radical and consequently a decrease in their reactivity. This finding is consistent with the conclusions drawn in Section 3.5.1, which determined through bond dissociation energy calculations that “FQ molecules with a methoxy group connected at the 1-position of the basic skeleton are less likely to undergo piperazine ring cleavage reactions.”

Overall, the addition of a methoxy group at the 1-position of FQ molecules can cause a sparser distribution of the HOMO over the carbon atoms involved in the piperazine ring, defluorination, and hydroxylation reactions, thereby weakening the overall reactivity of FQ molecules with radicals. Moreover, the addition of a methoxy group at the 1-position leads to an increase in the HOMO-LUMO energy gap between FQ molecules and radicals, resulting in decreased reactivity of FQ molecules with the hydroxyl radical.

## 4. Conclusions

In this research, density functional theory (DFT) was employed to quantify the oxidative degradation potential of 16 commercially available fluoroquinolone (FQ) molecules within advanced oxidation systems. The study elucidates a significant correlation between the molecular structural characteristics of FQ molecules and their degradation capabilities across five identified advanced oxidation pathways. Additionally, three-dimensional quantitative structure–activity relationship (3D-QSAR) contour maps were utilized to discern the influence of specific functional groups on the advanced oxidation degradation of FQ molecules. Moreover, the study utilized VEGA software to evaluate the toxicity risks posed by FQs and their degradation by-products, proposing restrictions on FQ molecules that present a high risk and strategies to circumvent degradation reactions that could heighten the toxicity of degradation products. The intent of this research was to provide a data-driven foundation for thorough and rational health risk assessments of FQ molecules, to contribute novel perspectives for directing the efficient and sustainable degradation of FQs in advanced oxidation systems, and to offer theoretical support for the design of eco-friendly FQ alternatives.

## Figures and Tables

**Table 1:** Calculation of bond dissociation energies during the primary degradation process of FQ molecules in advanced oxidation systems.

FQ Molecule	Bond Dissociation Energy (kJ/mol)						
	Bond Dissociation Energy 1	Bond Dissociation Energy 2	Bond Dissociation Energy 3	Bond Dissociation Energy 4	Bond Dissociation Energy 5	Average Bond Dissociation Energy 2, 3, 5	Average Bond Dissociation Energy 1–5
CIP	315.215	475.796	428.048	345.621	420.067	441.304	396.949
CLI	-	476.809	427.754	-	417.685	440.750	-
ENO	332.520	469.497	435.749	354.007	419.707	441.651	402.296
FLE	323.916	478.663	432.779	356.372	419.095	443.513	402.165
GAT	345.581	478.644	436.300	355.487	419.518	444.821	407.106
LEV	323.651	480.971	424.094	362.482	419.069	441.378	402.053
LOM	302.263	468.812	421.597	335.379	420.174	436.861	389.645
MOX	-	474.677	430.099	-	420.721	441.832	-
NAD	-	485.717	434.137	-	422.396	447.417	-
NOR	334.738	473.383	420.770	343.804	421.537	438.563	398.846
OFL	285.316	472.396	424.136	338.739	418.284	438.272	387.774
PEF	332.362	478.256	433.633	355.115	420.883	444.257	404.050
RUF	322.860	483.039	435.182	354.429	420.621	446.281	403.226
SPA	329.582	479.695	435.563	359.746	424.528	446.595	405.822
TOS	-	481.703	439.522	-	418.397	446.541	-
GEM	-	477.578	434.822	-	417.869	443.423	-

**Table 2:** CoMSIA model evaluation parameters for the bond dissociation energies of FQ molecules in advanced oxidation systems.

CoMSIA	*q* ^2^	*N*	*R* ^2^	*SEE*	*F*	*r* ^2^_pred_	*SEP*
Piperazine ring cleavage	0.516	5	1.000	0.001	227534.936	0.972	0.043
Defluorination	0.595	6	1.000	0.002	26018.716	0.992	0.062
Hydroxylation	0.628	7	1.000	0.008	1926.276	0.878	0.018
Piperazine ring hydroxylation	0.622	2	0.991	0.037	268.732	0.999	0.006

**Table 3:** Molecular force field contributions of the CoMSIA model for the bond dissociation energies of FQ molecules in advanced oxidation systems.

CoMSIA	Hydrogen Bond Acceptor Field (%)	Hydrophobic Field (%)	Electrostatic Field (%)	Hydrogen Bond Donor Field (%)	Steric Field (%)
Piperazine ring cleavage	49.30	21.00	15.40	7.30	7.00
Defluorination	43.20	22.30	18.20	8.60	7.80
Hydroxylation	25.30	24.70	24.50	6.20	19.20
Piperazine ring hydroxylation	40.60	16.90	28.50	7.70	6.30

**Table 4:** Feature importance of FQ molecular feature parameters selected by decision tree screening algorithm.

Implicit Variable	Feature Parameter	Feature Importance
Bond dissociation energy 1	(AlogP)^2^	0.713
	MATS2m	0.187
	maxsssCH	0.069
	GATS5c	0.022
	ATS6s	0.005
	MATS1m	0.002
	SP-2	0.002
	SM1_Dze	<0.001
	AVP-1	<0.001
Bond dissociation energy 2	ECCEN	0.737
	MATS8i	0.132
	MATS3c	0.065
	nHaaCH	0.006
	SpMin3_Bhm	0.003
	SpMin8_Bhi	0.001
	EE_Dzp	<0.001
	MAXDP	<0.001
Bond dissociation energy 3	ALogp2	0.847
	GATS6e	0.105
	SP-2	0.023
	GATS3i	0.001
	SpMAD_Dt	<0.001
	GATS5i	<0.001
	C1SP2	<0.001
Bond dissociation energy 4	MLFER_BH	0.749
	SP-2	0.119
	GATS6i	0.085
	Sare	0.037
	SpMax8_Bhe	0.005
	SP-5	0.003
	GATS6e	0.002
	ATS0m	<0.001
	ATS3i	<0.001

## Data Availability

Data are available upon request.

## Supplementary Materials

The following supporting information can be downloaded at https://www.mdpi.com/article/10.3390/toxics12030203/s1: Figure S1: Equipotential diagram of FQs molecule CoMSIA model (a) hydrogen bond receptor field of defluorinated CoMSIA model; (b) Hydrophobic field of defluorinated CoMSIA model; (c) Defluorinated CoMSIA model electrostatic field; (d) Defluorinated CoMSIA model hydrogen bond donor field; (e) Defluorinated CoMSIA model three-dimensional field; (f) Hydroxylated CoMSIA model hydrogen bond receptor field; (g) Hydrophobic field of hydroxylated CoMSIA model; (h) Hydroxylated CoMSIA model electrostatic field; (i) Hydroxylated CoMSIA model hydrogen bond donor field; (j) Hydroxylated CoMSIA model stereo field; (k) CoMSIA model hydrogen bond receptor field for hydroxylation of piperazine ring; (l) Hydrophobic field of CoMSIA model for hydroxylation of piperazine ring; (m) CoMSIA model electrostatic field for hydroxylation of piperazine ring; (n) CoMSIA model hydrogen bond donor field for hydroxylation of piperazine ring; (o) CoMSIA model for hydroxylation of piperazine ring in three-dimensional field; Figure S2: FQs Molecular column clustering analysis chart; Table S1: The risk assessment of human health and the ecological impact of these FQ molecules and their intermediates; Table S2: Nucleophilic attack, electrophilic attack and free radical attack index of different FQs molecules; Table S3: LUMO-HOMO between different FQs molecules and hydroxyl radicals.

## References

[1] Li S., Wu Y., Zheng H., Li H., Zheng Y., Nan J., Chang J.S. (2023). Antibiotics degradation by advanced oxidation process (AOPs): Recent advances in ecotoxicity and antibiotic-resistance genes induction of degradation products. Chemosphere.

[2] Focazio M.J., Kolpin D.W., Barnes K.K., Furlong E.T., Meyer M.T., Zaugg S.D., Thurman M.E. (2008). A national reconnaissance for pharmaceuticals and other organic wastewater contaminants in the United States—II) Untreated drinking water sources. Sci. Total Environ..

[3] Tello A., Austin B., Telfer T.C. (2012). Selective pressure of antibiotic pollution on bacteria of importance to public health. Environ. Health Perspect..

[4] Rosal R., Rodríguez A., Perdigón-Melón J.A., Petre A., García-Calvo E., Gómez M.J., Fernández-Alba A.R. (2010). Occurrence of emerging pollutants in urban wastewater and their removal through biological treatment followed by ozonation. Water Res..

[5] Larsson D.J., de Pedro C., Paxeus N. (2007). Effluent from drug manufactures contains extremely high levels of pharmaceuticals. J. Hazard. Mater..

[6] Akbari M.Z., Xu Y., Lu Z., Peng L. (2021). Review of antibiotics treatment by advance oxidation processes. Environ. Adv..

[7] Wang J., Zhuan R. (2020). Degradation of antibiotics by advanced oxidation processes: An overview. Sci. Total Environ..

[8] Wang J., Wang S. (2018). Microbial degradation of sulfamethoxazole in the environment. Appl. Microbiol. Biotechnol..

[9] Wang J., Wang S. (2019). Preparation, modification and environmental application of biochar: A review. J. Clean. Prod..

[10] Zhuang S., Liu Y., Wang J. (2020). Covalent organic frameworks as efficient adsorbent for sulfamerazine removal from aqueous solution. J. Hazard. Mater..

[11] Avramiotis E., Frontistis Z., Manariotis I.D., Vakros J., Mantzavinos D. (2021). On the performance of a sustainable rice husk biochar for the activation of persulfate and the degradation of antibiotics. Catalysts.

[12] Wang X., Brigante M., Dong W., Wu Z., Mailhot G. (2020). Degradation of Acetaminophen via UVA-induced advanced oxidation processes (AOPs). Involvement of different radical species: HO, SO^4−^ and HO^2^/O^2−^. Chemosphere.

[13] Bermúdez L.A., Pascual J.M., Martínez M.D.M.M., Poyatos Capilla J.M. (2021). Effectiveness of advanced oxidation processes in wastewater treatment: State of the art. Water.

[14] Sciscenko I., Hắng H.T.M., Escudero-Oñate C., Oller I., Arques A. (2021). Fluorescence spectroscopy and chemometrics: A simple and easy way for the monitoring of fluoroquinolone mixture degradation. ACS Omega.

[15] Ao X., Zhang X., Li S., Yang Y., Sun W., Li Z. (2023). Comprehensive understanding of fluoroquinolone degradation via MPUV/PAA process: Radical chemistry, matrix effects, degradation pathways, and toxicity. J. Hazard. Mater..

[16] Bobu M., Yediler A., Siminiceanu I., Zhang F., Schulte-Hostede S. (2013). Comparison of different advanced oxidation processes for the degradation of two fluoroquinolone antibiotics in aqueous solutions. J. Environ. Sci. Health A.

[17] Li M., Wei D., Zhao H., Du Y. (2014). Genotoxicity of quinolones: Substituents contribution and transformation products QSAR evaluation using 2D and 3D models. Chemosphere.

[18] Wang D., Ning Q., Dong J., Brooks B.W., You J. (2020). Predicting mixture toxicity and antibiotic resistance of fluoroquinolones and their photodegradation products in *Escherichia coli*. Environ. Pollut..

[19] Wachter N., Aquino J.M., Denadai M., Barreiro J.C., Silva A.J., Cass Q.B., Rocha-Filho R.C. (2019). Electrochemical degradation of the antibiotic ciprofloxacin in a flow reactor using distinct BDD anodes: Reaction kinetics, identification and toxicity of the degradation products. Chemosphere.

[20] Anjali R., Shanthakumar S. (2019). Insights on the current status of occurrence and removal of antibiotics in wastewater by advanced oxidation processes. J. Environ. Manage..

[21] Aydogdu S., Hatipoglu A. (2023). Aqueous degradation of 6-APA by hydroxyl radical: A theoretical study. J. Mol. Model..

[22] Wang Z., Pu Q., Li Y. (2023). Bidirectional selection of the functional properties and environmental friendliness of organophosphorus (OP) pesticide derivatives: Design, screening, and mechanism analysis. Sci. Total Environ..

[23] Munkhdalai L., Munkhdalai T., Park K.H., Lee H.G., Li M., Ryu K.H. (2019). Mixture of activation functions with extended min-max normalization for forex market prediction. IEEE Access.

[24] Clark M., Cramer R.D., Van Opdenbosch N. (1989). Validation of the general purpose tripos 5.2 force field. J. Comput. Chem..

[25] Pu Q., Han Z., Li X., Li Q., Li Y. (2022). Designing and screening of fluoroquinolone substitutes using combined in silico approaches: Biological metabolism–bioconcentration bilateral selection and their mechanism analyses. Green Chem..

[26] Li Q., Qiu Y., Li Y. (2020). Molecular design of environment-friendly PAE derivatives based on 3D-QSAR assisted with a comprehensive evaluation method combining toxicity and estrogen activities. Water Air Soil Pollut..

[27] Liu Y., Li X., Pu Q., Fu R., Wang Z., Li Y., Li X. (2023). Innovative screening for functional improved aromatic amine derivatives: Toxicokinetics, free radical oxidation pathway and carcinogenic adverse outcome pathway. J. Hazard. Mater..

[28] Haghighatlari M., Hachmann J. (2019). Advances of machine learning in molecular modeling and simulation. Curr. Opin. Chem. Eng..

[29] Dong Y., Georgakis C., Santos-Marques J., Du J. (2021). Dynamic response surface methodology using Lasso regression for organic pharmaceutical synthesis. Front. Chem. Sci. Eng..

[30] Patel P.S., Pandya D.M., Shah M. (2023). A holistic review on the assessment of groundwater quality using multivariate statistical techniques. Environ. Sci. Pollut. Res..

[31] Zhao Y.Y., Li Y. (2019). Design of environmentally friendly neonicotinoid insecticides with bioconcentration tuning and Bi-directional selective toxic effects. J. Clean. Prod..

[32] Salahinejad M., Ghasemi J.B. (2014). 3D-QSAR studies on the toxicity of substituted benzenes to Tetrahymena pyriformis: CoMFA, CoMSIA and VolSurf approaches. Ecotoxicol. Environ. Saf..

[33] Segalin J., Arsand J.B., Jank L., Schwalm C.S., Streit L., Pizzolato T.M. (2022). In silico toxicity evaluation for transformation products of antimicrobials, from aqueous photolysis degradation. Sci. Total Environ..

[34] Drgoňa J., Tuor A.R., Chandan V., Vrabie D.L. (2021). Physics-constrained deep learning of multi-zone building thermal dynamics. Energy Build..

[35] Khalifa N.M., Srour A.M., Abd El-Karim S.S., Saleh D.O., Al-Omar M.A. (2017). Synthesis and 2D-QSAR study of active benzofuran-based vasodilators. Molecules.

[36] Ghose A.K., Pritchett A., Crippen G.M. (1988). Atomic physicochemical parameters for three dimensional structure directed quantitative structure-activity relationships III: Modeling hydrophobic interactions. J. Comput. Chem..

[37] Liu P., Wu Z., Lee J., Cravotto G. (2024). Sonocatalytic degrading antibiotics over activated carbon in cow milk. Food Chem..

[38] Sharma V., Goswami R., Madan A.K. (1997). Eccentric connectivity index: A novel highly discriminating topological descriptor for structure− property and structure− activity studies. J. Chem. Inf. Comput. Sci..

[39] Tian Z., Li G., Bai M., Hou X., Li X., Zhao C., Zhu Q., Du C., Li M., Liu W. (2022). Microbial mechanisms of refractory organics degradation in old landfill leachate by a combined process of UASB-A/O-USSB. Sci. Total Environ..

[40] Zhu Y., Zhou X., Chen D., Li F., Xue T., Farag A.S. (2017). Ternary Fe 3 O 4@ PANI@ Au nanocomposites as a magnetic catalyst for degradation of organic dyes. Sci. China Technol. Sci..

[41] Platts J.A., Butina D., Abraham M.H., Hersey A. (1999). Estimation of molecular linear free energy relation descriptors using a group contribution approach. J. Chem. Inf. Comput. Sci..

[42] Pucci R., Angilella G.G.N. (2022). Density functional theory, chemical reactivity, and the Fukui functions. Found. Chem..

[43] Bao C., Zhao J., Sun Y., Zhao X., Zhang X., Zhu Y., Xing B. (2021). Enhanced degradation of norfloxacin by Ce-mediated Fe-MIL-101: Catalytic mechanism, degradation pathways, and potential applications in wastewater treatment. Environ. Sci. Nano.

[44] Lu T., Chen F.W. (2012). Comparison of computational methods for atomic charges. Acta Phys.-Chim. Sin..

[45] Asghar A., Bello M.M., Raman A.A.A., Daud W.M.A.W., Ramalingam A., Zain S.B.M. (2019). Predicting the degradation potential of Acid blue 113 by different oxidants using quantum chemical analysis. Heliyon.

