# Supplementary material for: Identification and Mechanistic Analysis of Toxic Degradation Products in the Advanced Oxidation Pathways of Fluoroquinolone Antibiotics
Source: Toxics. 2024 Mar 6;12(3):203. doi: 10.3390/toxics12030203 (PMC10976194; doi:10.3390/toxics12030203)
Supplement: Supplementary file 1 [file toxics-12-00203-s001.zip › toxics-2868081-supplementary.pdf]

Supplementary Information

Identification and Mechanistic Analysis of Toxic Degradation Products in the Advanced Oxidation Pathways of Fluoroquinolone Antibiotics

Shuhai Sun [1,†], Zhonghe Wang [2,†], Qikun Pu [2], Xinao Li [2], Yuhan Cui [2], Hao Yang [2,*] and Yu Li [2,*]

[1] School of Hydraulic and Environmental Engineering, Changchun Institute of Technology,
Changchun 130012, China; sun18117471@163.com

[2] MOE Key Laboratory of Resources and Environmental System Optimization, North China Electric Power University, Beijing 102206, China; zhonghe_wang1999@163.com (Z.W.); puqikun2000@163.com (Q.P.); lixinao921734261@163.com (X.L.); 120222232050@ncepu.edu.cn (Y.C.)

* Correspondence: yh13601614368@163.com (H.Y.); liyuxx8@hotmail.com (Y.L.)

† These authors contributed equally to this work.

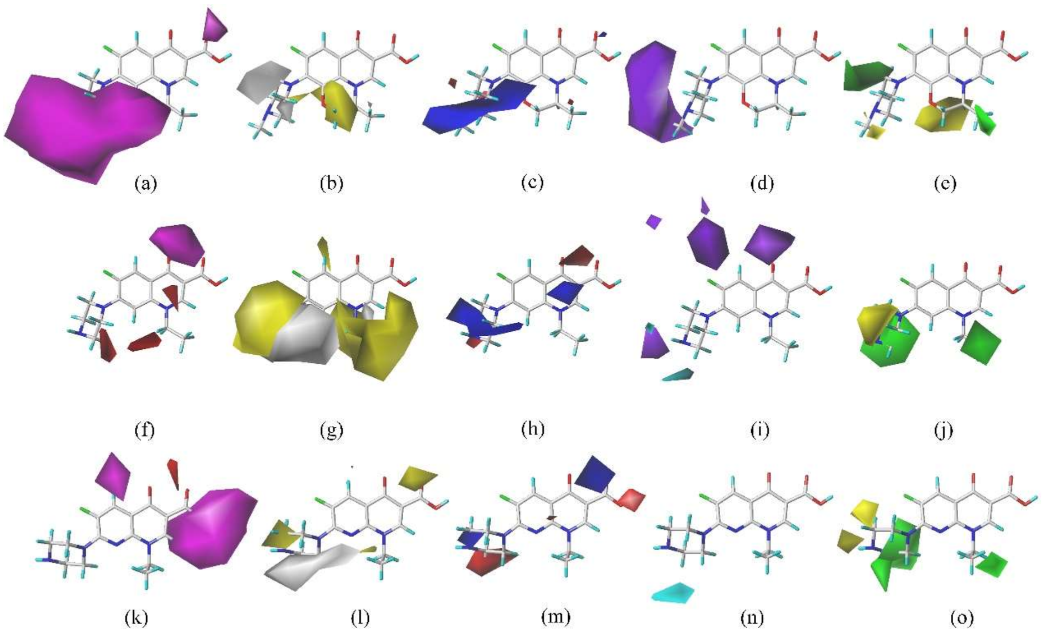

Figure S1 Equipotential diagram of FQs molecule CoMSIA model ((a) hydrogen bond receptor field of defluorinated CoMSIA model; (b) Hydrophobic field of defluorinated CoMSIA model; (c) Defluorinated CoMSIA model electrostatic field; (d) Defluorinated CoMSIA model hydrogen bond donor field; (e) Defluorinated CoMSIA model three-dimensional field; (f) Hydroxylated CoMSIA model hydrogen bond receptor field; (g) Hydrophobic field of hydroxylated CoMSIA model; (h) Hydroxylated CoMSIA model electrostatic field; (i) Hydroxylated CoMSIA model

hydrogen bond donor field; (j) Hydroxylated CoMSIA model stereo field; (k) CoMSIA model hydrogen bond receptor field for hydroxylation of piperazine ring; (l) Hydrophobic field of CoMSIA model for hydroxylation of piperazine ring; (m) CoMSIA model electrostatic field for hydroxylation of piperazine ring; (n) CoMSIA model hydrogen bond donor field for hydroxylation of piperazine ring; (o) CoMSIA model for hydroxylation of piperazine ring in three-dimensional field.

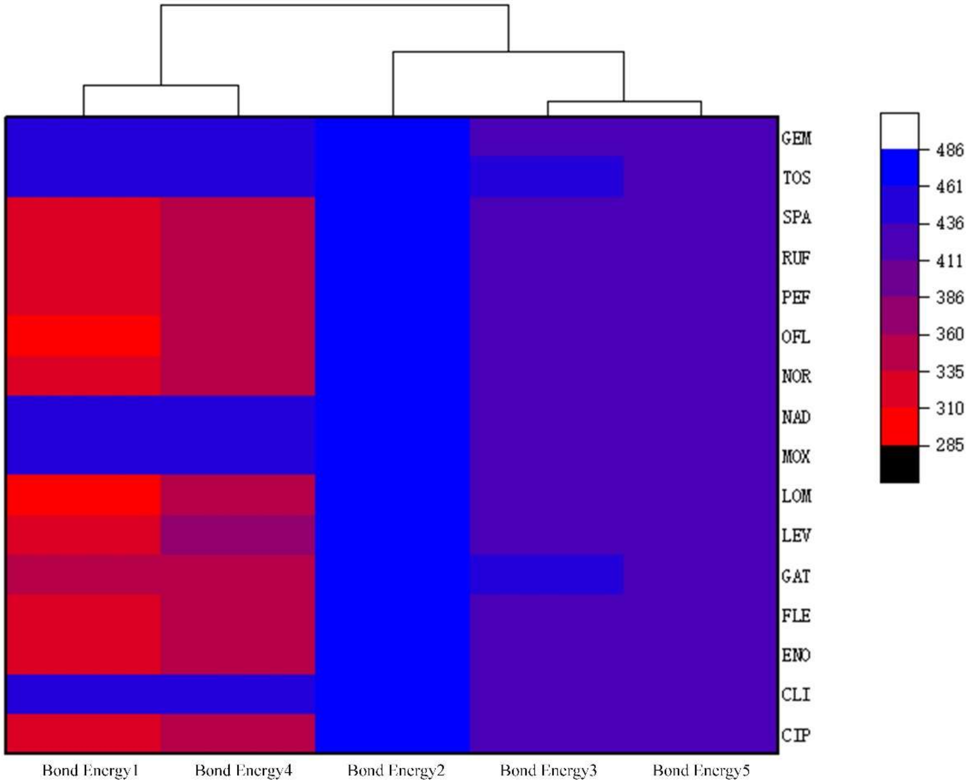

Figure S2 FQs Molecular column clustering analysis chart.
